# Characterization of β-*N*-acetylhexosaminidase (LeHex20A), a member of glycoside hydrolase family 20, from *Lentinula edodes* (shiitake mushroom)

**DOI:** 10.1186/2191-0855-2-29

**Published:** 2012-06-01

**Authors:** Naotake Konno, Hideyuki Takahashi, Masahiro Nakajima, Takumi Takeda, Yuichi Sakamoto

**Affiliations:** 1 Iwate Biotechnology Research Center, 22-174-4 Narita, Kitakami-shi, Iwate 024-0003, Japan

**Keywords:** β-*N*-acetylglucosaminide, Chitin, Fungal cell wall, Glycoside hydrolase family 20, Basidiomycete

## Abstract

We purified and cloned a β-*N*-acetylhexosaminidase, LeHex20A, with a molecular mass of 79 kDa from the fruiting body of *Lentinula edodes* (shiitake mushroom). The gene *lehex20a* gene had 1,659 nucleotides, encoding 553 amino acid residues. Sequence analysis indicated that LeHex20A belongs to glycoside hydrolase (GH) family 20, and homologues of *lehex20a* are broadly represented in the genomes of basidiomycetes. Purified LeHex20A hydrolyzed the terminal monosaccharide residues of β-*N*-acetylgalactosaminides and β-*N*-acetylglucosaminides, indicating that LeHex20A is a β-*N*-acetylhexosaminidase classified into EC 3.2.1.52. The maximum LeHex20A activity was observed at pH 4.0 and 50°C. The kinetic constants were estimated using chitooligosaccharides with degree of polymerization 2-6. GH20 β-*N*-acetylhexosaminidases generally prefer chitobiose among natural substrates. However, LeHex20A had the highest catalytic efficiency (*k*_cat_/*K*_m_) for chitotetraose, and the *K*_m_ values for GlcNAc_6_ were 3.9-fold lower than for chitobiose. Furthermore, the enzyme partially hydrolyzed amorphous chitin polymers. These results indicate that LeHex20A can produce *N*-acetylglucosamine from long-chain chitomaterials.

## Introduction

Chitin, composed of β-1,4 linked *N-*acetylglucosamine (GlcNAc) units, is mainly found in crustaceans, insects and fungi. Enzymatic degradation of chitin is catalyzed by a two-component chitinolytic enzyme system. One component is chitinases (EC 3.2.1.14), which hydrolyze β-1,4 linkages in chitin polymers, endolytically producing chitooligosaccharides, especially chitobiose (Brurberg et al. 1996; Tanaka et al. 2001). The other is β-*N*-acetylhexosaminidases (EC 3.2.1.52), which typically have no activity against chitin polymers, and instead degrade chitooligosaccharides formed by chitinases into monomers. Because the enzymes prefer short β-*N*-acetylglucosaminide substrates, chitobiose and p-nitrophenyl-*N*-acetyl-β-D-glucosaminide (pNP-GlcNAc), they are also called chitobiases (Drouillard et al. 1997; Tews et al. 1996). β-*N*-acetylhexosaminidases are widely distributed in animal tissues (Korneluk et al. 1986), insects (Hogenkamp et al. 2008; Yang et al. 2008), plants (Meli et al. 2010), bacteria (Clarke et al. 1995; Mark et al. 2001) and fungi (Cannon et al. 1994; López-Mondéjar et al. 2009; Jones and Kosman 1980), and belong to glycoside hydrolase (GH) families 3, 20 and 84 as categorized in the CAZy database (http://www.cazy.org/index.html). The GH20 enzymes hydrolyze nonreducing terminal monosaccharide residues of β-*N*-acetylgalactosaminides and β-*N*-acetylglucosaminides.

In fungi, chitin is a main cell-wall component, together with β-glucans (Iten and Matile 1970; Vetter 2007), and most filamentous fungi such as ascomycetes and basidiomycetes produce chitinolytic enzymes. Some mycoparasitic fungi such as *Trichoderma* species produce extracellular chitinolytic enzymes for degradation of host cell walls during their mycoparasitic attack (Carsolio et al. 1994; Peterbauer et al. 1996; Seidl et al. 2006). On the other hand, some fungal chitinolytic enzymes act on their own cell walls during changes in morphology, which are an essential process in the fungal cell cycle (Mitchell and Sabar 1966; Seiler and Plamann 2003). For example, some chitinases (Rast et al. 1991; Shin et al. 2009) and β-*N*-acetylhexosaminidases (Cannon et al. 1994; Kim et al. 2002) from filamentous fungi such as *Aspergillus* and *Mucor* species are suggested to have roles in processes such as hyphal autolysis, growth and branching. However, little information is known about the physiological function and role of fungal chitinolytic enzymes. Moreover, there have been no reports of cloned and characterized chitinolytic enzymes from basidiomycetes.

Most basidiomycetes form a fruiting body (mushroom) as part of their usual life cycle. The cell walls of the fruiting body are constructed mainly from chitin and β-glucans, and these polysaccharides are self-degraded by enzymes associated with cell walls during morphological changes (Shida et al. 1981; Minato et al. 2004). Recently, we reported identification of four β-1,3-glucanases (EXG1, EXG2, TLG1 and GLU1) and one β-1,6-glucanase (LePus30A) from the *Lentinula edodes* fruiting body, the shiitake mushroom (Sakamoto et al. 2005a; 2005b; 2006; 2011; Konno and Sakamoto 2011). While enzymes involved in cell wall metabolism of *L. edodes* have been reported only for those acting on β-glucan, the presence of chitinolytic enzymes were suggested in our previous study (Sakamoto et al. 2009). In the present study, we purified and characterized a β-*N*-acetylhexosaminidase, LeHex20A, from the fruiting body of *L. edodes*.

## Materials and methods

### Materials

*L. edodes* cultivated dikaryotic strain H600 (Hokken. Co., Ltd) was used in all experiments (Sakamoto et al., 2005a). Fruiting bodies for RNA and protein extraction were prepared using the method of Nagai et al. (2003). Mature fruiting bodies were separated into pileus, gill and stipe. Harvested mature fruiting bodies were immediately transferred to a desiccator at 25°C and 80% humidity for post-harvest preservation. All samples were stored at -80°C.

Colloidal chitin was prepared according to Hsu and Lockwood (1975). Mechanochemically ground chitin was kindly provided by the Department of Chemical Engineering, Ichinoseki National College of Technology (Nakagawa et al. 2011).

### Purification of β-*N*-acetylhexosaminidase

Proteins were extracted from 320 g of fresh fruiting bodies. Samples were crushed in liquid nitrogen, suspended in 320 ml of 10 mM sodium phosphate buffer (pH 7.0), and incubated with rotation for 30 min at room temperature. Ammonium sulfate was added until the concentration reached 70% saturation. The resulting precipitates were collected by centrifugation (30 min, 4,500 × *g*) and dissolved in 10 mM sodium phosphate buffer (pH 7.0) containing ammonium sulfate at 30% saturation. The supernatant was applied to a Phenyl-Toyopearl column (1.6 × 10 cm, Tosoh Co., Ltd., Tokyo, Japan) equilibrated with 10 mM sodium phosphate buffer (pH 7.0) containing ammonium sulfate at 30% saturation. The column was washed with 45 ml of the same buffer, and proteins were eluted in 45 ml of a linear concentration gradient (30-0% saturation) of ammonium sulfate at a flow rate of 1.5 ml/min. Fractions containing β-*N*-acetylhexosaminidase activity were collected and concentrated using an Amicon Ultra 5,000 NMWL filter (Millipore, Billerica, MA, USA), and then applied to a MonoQ 5/50 GL anion exchange column (0.5 × 5 cm, GE Healthcare, Little Chalfont, UK). Adsorbed proteins were eluted using a linear concentration gradient of NaCl (0–0.5 M) at a flow rate of 0.5 ml/min. The eluted enzyme was then applied to a DEAE-Toyopearl Pak 650S anion exchange column (0.8 × 7.5 cm, Tosoh Co., Ltd.) equilibrated with 10 mM sodium phosphate buffer. The enzyme was eluted with a linear concentration gradient of NaCl (60 ml, 0–0.5 M) at a flow rate of 0.5 ml/min. Fractions containing activity were collected and concentrated. Concentrated proteins were then applied to a Superdex 75 10/30 gel filtration column (GE Healthcare) equilibrated in 10 mM sodium phosphate buffer (pH 7.0) with 0.1 M NaCl, and proteins were eluted with the same buffer at a flow rate of 0.4 ml/min. Purified LeHex20A was analyzed by SDS-PAGE, and the N-terminal amino acid sequence of LeHex20A was analyzed as described in Sakamoto et al. (2005a).

### Cloning and sequencing of the *lehex20a* gene

cDNA was synthesized from total RNA extracted from fresh fruiting bodies using the SMART PCR RACE kit (BD Bioscience, CA, USA), according to the manufacturer’s protocol. 3′-RACE was performed using degenerate primers (chi4-3U: 5′-ACN GYN GYN ATG GTN TGG AT-3′ and chi4-4U: 5′-TGG TGY GAY CCN TTY AAR AC-3′) designed against conserved amino acid sequences of GH family 20 in filamentous fungi. cDNA for the 5′-RACE PCR template was synthesized from the RNA using a GeneRacer kit (Invitrogen, CA, USA), and PCR was performed as described previously (Sakamoto et al., 2005b) using specific primers (chi4-56-RACEL: 5′-AGT TTA GCT TGA GCA TCA GTC AAA T-3′ and chi4-93-RACEL: 5′-CTC GGT CCA AAG TAG GTG TTC T-3′) and GeneRacer primers (Invitrogen). The presence of a signal peptide in the deduced amino sequence was predicted using the SignalP server (http://www.cbs.dtu.dk/services/SignalP/). Comparative analysis of homology with enzymes registered in the GenBank databases was carried out using an NCBI BLAST search (http://www.ncbi.nlm.nih.gov/BLAST) with the default parameters.

### Enzyme assays

β-*N*-Acetylhexosaminidase activity was assayed in 20 mM sodium acetate buffer (pH 4.2) at 37°C for 15 min. For purification, activity was determined using 0.32 mM of 4-methylumbelliferyl β-D-*N,N',N"*-triacetylchitotrioside (4MU-GlcNAc_3_, an analogue of the natural substrate, GlcNAc_4_; Sigma-Aldrich Inc., St. Louis, MO, USA) as substrate (Hood 1991). The reaction was quenched with 0.4 M Na_2_CO_3_, and the released 4MU was measured by spectrophotofluorimetry with excitation at 365 nm and emission at 445 nm. The effects of pH (pH 3-9) and temperature (10-80°C) on enzyme activity were analyzed as described previously (Konno and Sakamoto 2011). To elucidate the substrate specificity of the enzyme, assays were performed using the following substrates: p-nitrophenyl-*N*-acetyl-β-D-glucosaminide (pNP-GlcNAc), p-nitrophenyl-*N*-acetyl-beta-D-galactosaminide (pNP-GalNAc), p-nitrophenyl-D-glucoside (pNP-Glc) (Sigma-Aldrich), chitooligosaccharides, (GlcNAc_2-6_, Seikagaku Biobusiness Co., Tokyo, Japan), the complex N-glycan (GlcNAcβ-1,2Manα-1,6) (GlcNAcβ-1,2Manα-1,3) Manβ-1,4GlcNAcβ-1,4GlcNAc-PA (TaKaRa Bio Inc., Shiga, Japan), chitin (Wako Pure Chemicals Co., Osaka, Japan), ethylene glycol chitin (Seikagaku kogyo, Co., Tokyo, Japan), colloidal chitin and the mechanochemically ground chitin. To assay pNP, the amount of pNP was determined spectrophotometrically at 405 nm. The extinction coefficient of pNP was assumed to be 17,100 M^-1^ cm^-1^. The amount of GlcNAc released from chitin oligomers and polymers was determined by the Morgan-Elson assay according to the method of Keyhani and Roseman (1996). One unit (U) of enzyme activity was defined as the amount of enzyme that produces 1 μmol GlcNAc per minute under the above conditions. To determine the kinetic properties of LeHex20A, the reactions were performed with 0.05-0.5 mM of substrate and 3.1 nM of purified LeHex20A in 20 mM sodium acetate buffer (pH 4.2) at 37°C for 15 min. In these reactions, the products formed from the chitooligosaccharides (GlcNAc_2-6_) were monosaccharides (GlcNAc) and oligosaccharides shortened by one GlcNAc unit (HPLC analyses, data not shown). The values of *k*_cat_ and *K*_m_ were estimated using Lineweaver-Burk plots.

### HPLC analysis

To study the degradation of natural substrates, 2% (w/v) amorphous chitins (colloidal chitin or mechanochemically grinded chitin) or 0.5 mM chitooligosaccharides were incubated at 30 °C in 20 mM sodium acetate buffer (pH 4.2), using 0.92 nM purified LeHex20A for the chitins and 0.40 nM LeHex20A for the chitooligosaccharides. The hydrolysis products were analyzed using an HPLC system equipped with a TSKgel Amide-80 column (4.6 × 250 mm, Tosoh). The mobile phase was 65% (v/v) acetonitrile at a flow rate of 1.0 ml/min, and the column temperature was 80°C. Eluted carbohydrates were detected by monitoring UV absorption at 205 nm.

### Nucleotide sequence accession number

The nucleotide sequence encoding LeHex20A has been deposited in the DDBJ/EMBL/GenBank databases under the accession number [DDBJ: AB703443].

## Results

### Purification of LeHex20A and cloning of its gene, *lehex20a*

**Figure 1:**
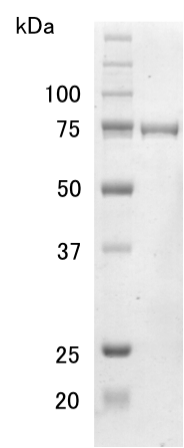
**SDS-PAGE of purified LeHex20A.** Approximately 1 μg of sample was separated on a 10% (w/v) polyacrylamide gel. Lane 1, molecular mass standards (kDa); lane 2, purified LeHex20A.

LeHex20A was purified from fresh fruiting bodies of *L. edodes* by four steps of column chromatography, with monitoring of activity towards 4MU-GlcNAc_3_. As a result of the purification, a single major band was obtained by SDS-PAGE, and the deduced molecular mass of the purified LeHex20A was 79 kDa (Figure 1). The N-terminal amino acid sequence of the 79 kDa protein was LWPLPTDFSTGTAAL, which was highly similar to the N-terminal amino acid sequence of GH family 20 (GH20) members in filamentous fungi. Because amplification by 3′-RACE PCR using primers designed based on the N-terminal sequence was unsuccessful, we designed degenerate primers based on the conserved amino acid sequence of GH20 members in filamentous fungi. We succeeded in amplifying a DNA fragment by 3′-RACE PCR, which contained a highly similar sequence to other GH20 members. Following 5′-RACE, we obtained full-length sequence for *lehex20a,* which contained a putative N-terminal amino acid sequence identical to the LeHex20A N-terminus. The cDNA contained an open reading frame of 1,659 bp, encoding 553 amino acid residues.

According to the results of SignalP analysis, the first 17 amino acid residues in the N-terminal region are expected to be a signal peptide, indicating that the mature protein, consisting of 536 amino acids, is an extracellular or cell wall protein. LeHex20A has a calculated molecular mass of 58 kDa, suggesting that the protein is glycosylated. Indeed, the amino acid sequence had 13 possible *N*-glycosylation sites (Asn-Xxx-Thr/Ser http://www.cbs.dtu.dk/services/NetNGlyc/). In addition, there were many possible sites for *O*-glycosylation (http://www.cbs.dtu.dk/services/NetOGlyc/). The sequence was analyzed using searches on the Pfam database (http://pfam.sanger.ac.uk/search/sequence). Search results for the sequence showed that the amino acid sequence contains GH20 domains. The deduced amino acid sequence was analyzed using the blastp algorithm of the NCBI protein database. The BLAST search showed that the sequence had up to 61% sequence identity to putative GH20 proteins (containing putative β-*N*-acetylhexosaminidase sequences) from basidiomycetous species such as *Serpula lacrymans* (EGN97893), *Coprinopsis cinerea* (XP_001835638) and *Postia placenta* (XP_002472465). The sequence showed homology to putative GH20 proteins from ascomycetes such as *Leptosphaeria maculans* (CBX95932) and *Trichoderma reesei* (EGR50812), although in these cases, sequence identity levels were only about 30%. These results suggest that LeHex20A belongs to GH20. Further sequence analyses were carried out using the blastp algorithm in genome sequence databases of basidiomycetes (http://genome.jgi-psf.org/programs/fungi/index.jsf). These searches revealed that LeHex20A has high levels of similarity to proteins of basidiomycetes including *Fomitopsis pinicola* (ID number from DOE Joint Genome Institute, 129075; similarity, 61%)*, Heterobasidion annosum* (151266; 58%)*, Agaricus bisporus* (120598; 58%) and *Pleurotus ostreatus* (57387/87509; 58%). Thus, homologues of *lehex20a* seem to be conserved in basidiomycetes.

### Enzymatic properties of LeHex20A

Effects of pH and temperature on enzyme activity were examined using 4MU-GlcNAc_3_ as a substrate. The maximum LeHex20A activity was observed at pH 4.0 in 20 mM sodium acetate buffer and at 50°C. The enzyme was stable across a pH range from 5 to 8 when incubated at 4°C for 20 h. The enzyme was inactivated after incubation at 60°C for 30 min.

Substrate specificity of the purified LeHex20A was estimated. In pNP-substrate assays, LeHex20A showed hydrolytic activity for not only pNP-GlcNAc, but also pNP-GalNAc, and no activity was detected for pNP-Glc. These results indicate that LeHex20A is a β-*N*-acetylhexosaminidase classified into EC 3.2.1.75. While some β-*N*-acetylhexosaminidases also cleave β-1,2 linkages in complex N-glycan substrates, resulting in liberation of terminal GlcNAc, LeHex20A had no activity for this type of substrate. When pNP-GlcNAc, pNP-GalNAc and chitooligosaccharides (GlcNAc_2-6_) were used as substrates, the kinetic constants, *k*_cat_/*K*_m_, were estimated as follows: GlcNAc_4_ > GlcNAc_3_ > GlcNAc_5_ > GlcNAc_6_ > pNP-GlcNAc > GlcNAc_2_ > pNP-GalNAc (Table 1). LeHex20A had high *k*_cat_ toward short-chain substrates, such as pNP-GlcNAc and GlcNAc_2_. The *k*_cat_ value for GlcNAc_2_ was 2.0-fold than for GlcNAc_4_ and 2.5-fold higher than for GlcNAc_6_. In contrast, the enzyme showed higher affinity for the longer-chain chitooligosaccharides: the *K*_m_ value for GlcNAc_4_ was 5.4-fold lower than for GlcNAc_2_ and GlcNAc_6_ was 3.9-fold lower.

**Table 1:** Kinetic parameters of LeHex20A

**Substrate**	***K***_**m**_**(mM)**	***k***_**cat**_**(s-1)**	***k***_**cat**_**/*K***_**m**_
pNP-GlcNAc	0.34 ± 0.01	335 ± 10	983
pNP-GalNAc	0.43 ± 0.03	177 ± 8	411
Chitobiose(DP2)	0.42 ± 0.04	242 ± 14	576.7
Chitotriose(DP3)	0.14 ± 0.001	236 ± 1	1667
Chitotetraose(DP4)	0.07 ± 0.008	119 ± 5	1689
Chitopentaose(DP5)	0.08 ± 0.001	97 ± 1	1190
Chitohexaose(DP6)	0.1 ± 0.002	97 ± 1	992

**Figure 2:**
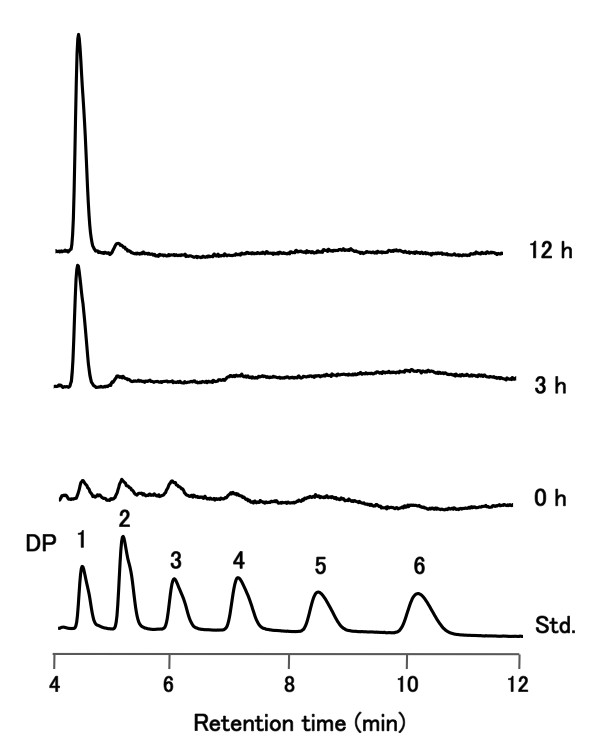
**HPLC analysis of LeHex20A action on colloidal chitin.** Colloidal chitin (2%, w/v) was incubated with LeHex20A (0.4 nM) in 20 mM sodium acetate buffer (pH 4.2), at 30°C. Eluted products were detected by monitoring UV absorption at 205 nm. Standards (Std.) were GlcNAc and chitooligosaccharides (GlcNAc_2-6_). DP; degree of polymerization.

LeHex20A activities for chitin polysaccharides including crystalline chitin, colloidal chitin, mechanochemically ground chitin and ethylene glycol chitin were examined. No activity was detected for crystalline chitin or ethylene glycol chitin. LeHex20A partially hydrolyzed colloidal chitin and mechanochemically ground chitin, although original insoluble chitin accumulated even after a long incubation with the enzyme. The specific activities determined by the rate of monomer production were 46.3 U/mg for colloidal chitin and 39.9 U/mg for mechanochemically ground chitin. To elucidate LeHex20A action against colloidal chitin, the supernatant in each reaction mixture was analyzed using HPLC (Figure 2). Chromatograms of samples at the start of the reaction showed the presence of various oligomers in the colloidal chitin, and these oligomers disappeared after 3 hours, accompanied by production of GlcNAc. In chromatograms of 12 h samples, more GlcNAc was produced as the reaction product. A similar result was obtained upon analyzing reaction products obtained from mechanochemically ground chitin (data not shown). These observations suggest that LeHex20A has exochitinase activity against amorphous chitin polysaccharides.

## Discussion

Knowledge of chitinolytic enzymes from basidiomycetes is important because these enzymes are likely to play roles on the autolysis of cell-wall chitin, which may result in morphological changes and affect the product quality of fruiting bodies (Kamada et al. 1982; Kües 2000; Mitchell and Sabar 1966; Sakamoto et al. 2009; Sone and Misaki 1978). Nevertheless, little is known about chitinolytic enzymes from basidiomycetes. In this study, we purified a protein with β-*N*-acetylhexosaminidase activity from fresh *L. edodes* fruiting bodies, and the analysis of its primary structure showed that the enzyme belongs to GH20. Therefore, we named the protein LeHex20A. To the best of our knowledge, this is the first report of a gene encoding a GH20 β-*N*-acetylhexosaminidase from basidiomycetes.

**Figure 3:**
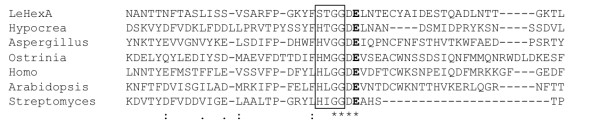
**Multiple sequence alignment of a region surrounding the catalytic residue of GH family 20 members (β-*N*-acetylhexosaminidases) from*Lentinula edodes*(LeHex20A),*Hypocrea lixii*(AAB50829),*Aspergillus oryzae*(XP_001817681),*Ostrinia furnacalis*(ABI81756),*Homo sapiens*(AAB00965),*Arabidopsis thaliana*(BAE99290) and*Streptomyces plicatus*(AAC38798).** Protein sequences were aligned with the MAFFT program (version 6; http://align.bmr.kyushu-u.ac.jp/mafft/online/server/) using the E-INS-I algorithm. A consensus motif preceding the catalytic residue is *boxed*. The catalytic glutamate is *bold*.

GH20 enzymes are retaining GHs and have catalytic domains with a (β/α)_8_ (TIM barrel) fold (Tews et al. 1996). The enzymes carry out substrate-assisted catalysis, in which a glutamate (Glu303 in LeHex20A; Figure 3) acts as the catalytic acid/base and the acetamido group of the sugar bound at the -1 subsite acts as a nucleophile (Drouillard et al. 1997; Jones and Kosman 1980). A conserved Asp adjacent to the catalytic acid (Asp302 in LeHex20A) is crucial for activating the acetamido group (Williams et al. 2002). While this catalytic dyad is conserved in LeHex20A, the consensus H-x-G-G motif, preceding the catalytic residue of representative GH20 members from bacteria, plant, insects, mammals, and ascomycetes, is not conserved in LeHex20A (Intra et al. 2008; Mayer et al. 2006). In LeHex20A and other basidiomycetes including *S. lacrymans, C. cinerea, P. placenta, F. pinicola, H. annosum, A. bisporus* and *P. ostreatus*, this motif has the sequence S-x-G-G, a sequence that seems unique for basidiomycete GH20 enzymes.

Most of the GH20 β-*N*-acetylhexosaminidases have the highest catalytic efficiency for GlcNAc_2_ among natural substrates, and do not hydrolyze long-chain chitomaterials (Keyhani and Roseman 1996; Koga et al. 1996; Ueda and Arai 1992; Yang et al. 2008). However, the catalytic efficiency of LeHex20A for GlcNAc_6_ was greater than for GlcNAc_2_, due to its high affinity for GlcNAc_6_. Moreover, the enzyme partially hydrolyzed two kinds of amorphous chitin polymers, resulting in production of GlcNAc. There are few reports about degradation of chitin polymers by β-*N*-acetylhexosaminidases (Suginta et al. 2010). Indeed, only six β-*N*-acetylhexosaminidases (EC 3.2.1.52) that are able to degrade chitin polymer are listed in the BRENDA database (http://www.brenda-enzymes.info/index.php4). Thus, LeHex20A showed unique enzymatic properties with respect to typical GH20 β-*N*-acetylhexosaminidases. Because β-*N*-acetylhexosaminidases hydrolyze nonreducing terminal monosaccharide residues of substrates, binding between the subsite (-1) of the enzymes and the nonreducing terminal β-*N*-acetylhexosaminides of substrates seems to be essential for the catalysis. Suginta et al. (2010) reported that GH20 β-*N*-acetylhexosaminidases from the bacterium *Vibrio harveyi* show hydrolytic activity toward colloidal chitin, and suggested that the enzyme has a binding pocket containing four GlcNAc binding subsites. Therefore, LeHex20A might have other GlcNAc binding subsites, as suggested for this protein by Suginta et al.

As described in this report, LeHex20A can produce GlcNAc from longer-chain chitomaterials compared with typical β-*N*-acetylhexosaminidases. Moreover, the *L. edodes* fruiting body, the shiitake mushroom, is a very popular edible cultivated mushroom. Therefore, LeHex20A may be valuable for efficient and safe enzymatic production of GlcNAc from native chitin materials.

## Competing interests

The authors declare that they have no competing interests.
